# Primary pulmonary synovial sarcoma with calcification: A case report

**DOI:** 10.1111/1759-7714.13172

**Published:** 2019-08-19

**Authors:** Hironori Ishida, Takashi Fujino, Ryo Taguchi, Hiroyuki Nitanda, Hirozo Sakaguchi, Akitoshi Yanagihara, Ryuichi Yoshimura

**Affiliations:** ^1^ Department of General Thoracic Surgery Saitama Medical University International Medical Center Saitama Japan; ^2^ Department of Pathology Saitama Medical University International Medical Center Saitama Japan

**Keywords:** Calcification, gene, lung, sarcoma, synovial

## Abstract

The lung is the organ most commonly affected by primary synovial sarcoma. Intratumoral calcification is less common in this organ versus soft tissue. Meanwhile, the presence of calcification in a lung nodule reduces the risk of lung cancer. Here, we report a case of pulmonary synovial sarcoma which manifested as a nodule with calcification, depicted on computed tomography (CT). A 52‐year‐old asymptomatic male was referred to Saitama Medical University International Medical Center and CT revealed a well‐defined nodule (1.8 cm), with punctate and eccentric calcification in the right lower lobe. Enhanced CT and 18F‐fluorodeoxyglucose positron‐emission tomography suggested a malignant tumor, and surgery was performed. Histology provided a preliminary diagnosis of monophasic spindle‐cell synovial sarcoma with hyalinized collagen bands and calcifications. Genetically, the presence of the SYT‐SSX2 fusion gene was consistent with the features of this disease. We conclude that primary pulmonary synovial sarcoma should be listed as a differential diagnosis for solitary pulmonary nodules with calcification.

## Key points

**Significant findings of the study**: Primary pulmonary synovial sarcoma can manifest as a nodule with calcification shown on computed tomography, and gene analysis is useful in reaching a definitive diagnosis for this disease.

**What this study adds**: Primary pulmonary synovial sarcoma should be listed as a differential diagnosis for solitary pulmonary nodules with punctuate and eccentric calcification.

## Introduction

Synovial sarcomas predominantly arise in the deep soft tissue of the lower and upper extremities; however, they appear in almost all anatomic locations. The most commonly involved visceral organ is the lung. The name synovial sarcoma is described as a misnomer, owing to the lack of evidence regarding synovial differentiation.^1^, ^2^ In clinical practice, ≤30% of cases with soft tissue synovial sarcoma exhibit focal calcifications on radiographs or computed tomography (CT) images. However, few cases of common calcifications have previously been described in synovial sarcomas arising in the lung.^3^ Currently, the histological diagnosis of synovial sarcoma is corroborated through gene analysis. In this article, we report a case of primary pulmonary synovial sarcoma in which focal calcification was revealed on CT. Verifying the presence of the t (X;18) (p11;q11) chromosomal translocation was useful in confirming the presence of synovial sarcoma.

### Case report

**Figure 1:**
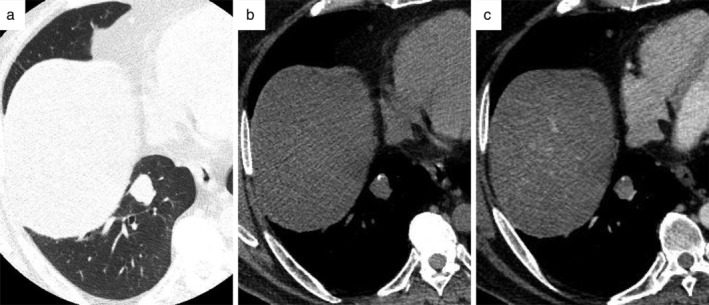
Diagnostic imaging. (**a**) Computed tomography (CT) with a lung window setting indicated the presence of a well‐defined solid nodule in the right lower lobe. (**b**) Noncontrast CT with a mediastinal window setting showed punctate calcification in the eccentric part of the nodule. (**c**) Contrast‐enhanced CT revealed a strong contrast enhancement of 50 Hounsfield units in the nodule.

During a follow‐up examination of post‐traumatic thoracic aortic aneurysm treated by a stent‐graft 17 years earlier, a 52‐year‐old asymptomatic male was referred to Saitama Medical University International Medical Center. The patient was undergoing treatment for hypertension, hyperlipidemia, and diabetes mellitus. Noncontrast chest CT incidentally revealed the presence of a well‐defined nodule measuring 1.8 cm, eccentrically with focal calcification in the right lower lobe (Fig 1(a,b)). On contrast‐enhanced CT, the nodule exhibited a heterogeneous enhancement pattern (Fig 1(c)). Chest X‐ray failed to show a nodule shadow due to an overlap with cardiac and phrenic shadows. Therefore, a bronchoscopic examination was not applicable. Blood analysis did not reveal significant findings related to tumor markers of lung cancer, or any evidence of mycobacterial and mycotic infections. A physical examination of the chest, abdomen, and extremities was unremarkable. 18F‐Fluorodeoxyglucose (FDG) positron‐emission tomography showed marginal FDG uptake, with a maximum‐standardized uptake value of 2.4 in the lung nodule. The examination excluded the presence of extrathoracic malignancies.

**Figure 2:**
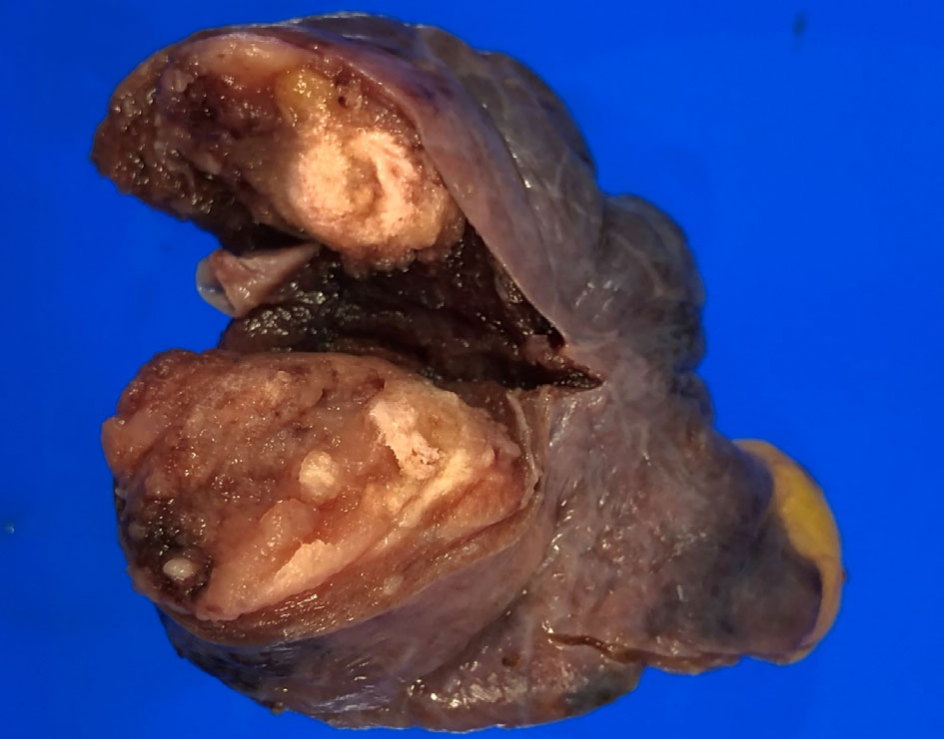
Macroscopic findings. The cut surface of the resected specimen showed a yellowish‐brown tumor with yellow‐whitish areas, measuring 1.8 cm in the largest diameter. The tumor was present entirely within the lung.

**Figure 3:**
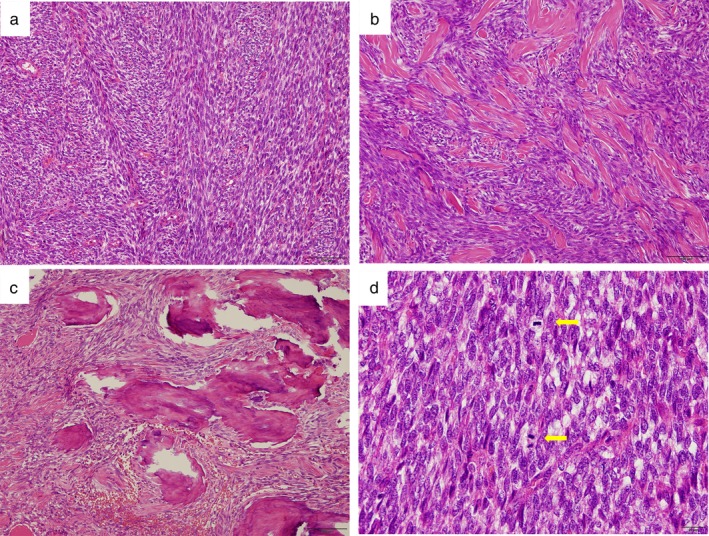
Histological findings using hematoxylin and eosin staining. (**a**) Spindle tumor cells are densely proliferating in interlacing fascicles with a herring‐bone pattern. (**b**) The tumor contained strands of ropy and wiry collagen, and bands of hyalinized collagen, corresponding to the yellow‐whitish areas on the cut surface (Fig. 2). (**c**) Foci of calcifications contiguous to the hyalinized collagen are shown. (**d**) Two mitoses of tumor cells are observed (arrow).

According to these findings, we considered the presence of a malignancy, although the presence of calcification within a lung nodule is generally not associated with primary lung cancer. Hence, we performed a wedge resection of the lung with the nodule, intraoperatively reaching a diagnosis of malignant spindle‐cell tumor. Subsequently, we performed right lower lobectomy with lymph node dissection. Grossly, the cut surface of the resected specimen showed a yellowish‐brown solid tumor with yellow‐whitish components, measuring 1.8 cm in the largest diameter (Fig 2). Histopathologically, the tumor was composed of spindle tumor cells arranged in interlacing fascicles or a herring‐bone pattern (Fig. 3(a)). Of note, epithelial features were lacking. Overall, the cellular portions of spindle cells alternated with less cellular areas, displaying hyalinized collagen bundles and calcifications (Fig. 3(b,c)). Mitoses ranged from four to six per 10 high‐power fields (Fig. 3(d)). Notably, the resected lymph nodes were not involved. Immunohistochemically, the tumor cells were negative for epithelial markers (epithelial membrane antigen and AE1/AE3), CD34, and Stat6. In contrast, the tumor cells were positive for CD99 and focally for S‐100. These findings provided a preliminary diagnosis of monophasic synovial sarcoma, which required differentiation from several other diseases belonging to spindle‐cell tumors.

**Figure 4:**
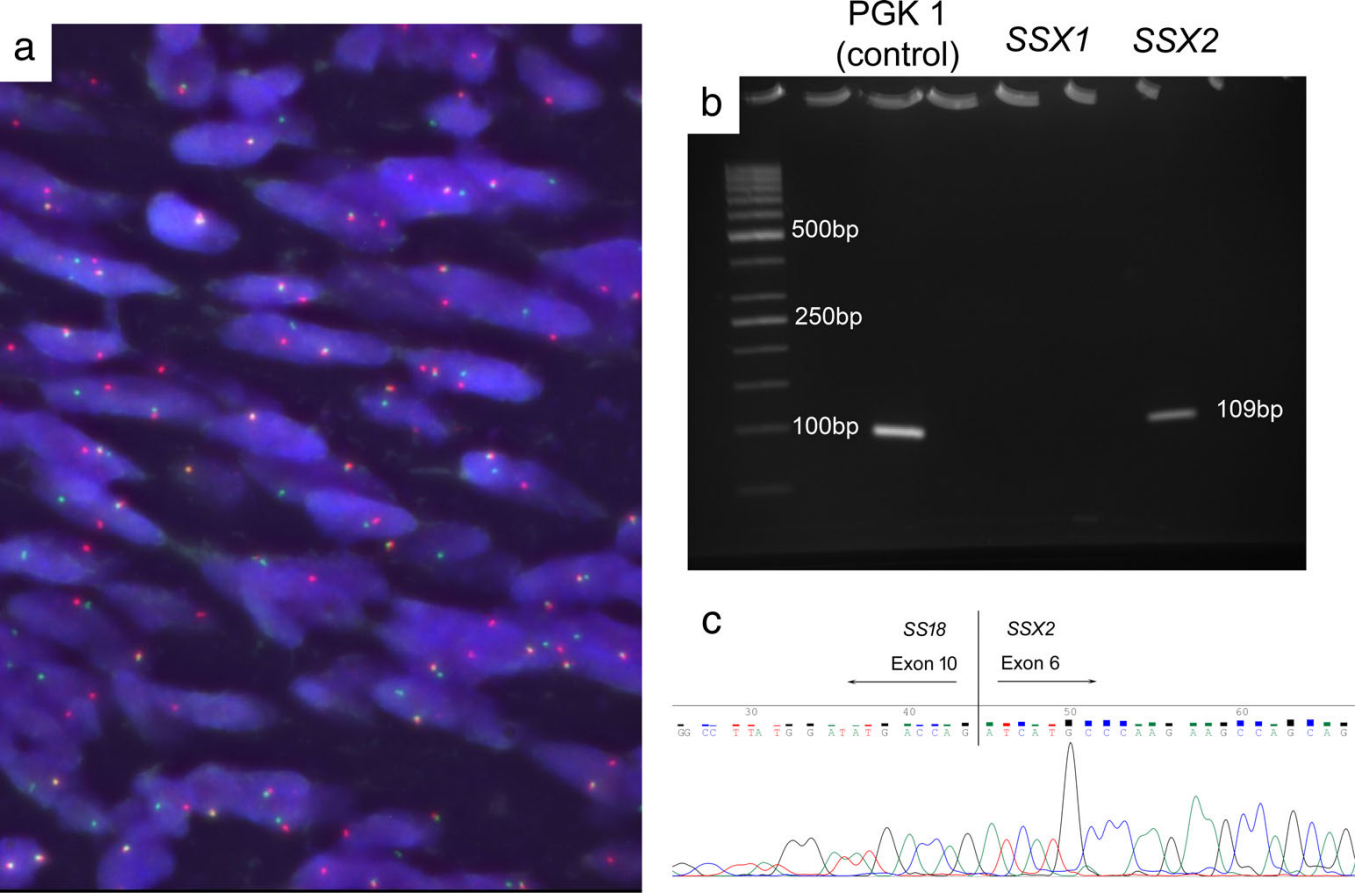
Gene analysis. (**a**) Fluorescence in situ hybridization analysis using formalin‐fixed, paraffin embedded tissues. A break‐apart signal with separate red and green signals revealed an *SS18* rearrangement, whereas two fused yellow signals did not demonstrate an *SS18* rearrangement. Almost all tumor cells revealed a *SS18* break‐apart signal, indicating a chromosomal translocation of the *SS18* gene. (**b**) Reverse transcription‐polymerase chain reaction analysis for the *SYT‐SSX* fusion gene. The *SYT‐SSX2* gene (109 bp) was amplified; however, the *SSX1* (151 bp) was negative. The housekeeping gene phosphoglycerokinase 1 (PGK 1: 100 bp), used as a positive control, was also amplified. (**c**) Sequencing analysis of the *SYT‐SSX* gene transcripts. A vertical line indicates the fusion site of the *SYT* and *SSX2* genes.

Genetically, fluorescence in situ hybridization analysis using a *SS18* break‐apart probe disclosed a rearrangement of the *SS18* gene (Fig. 4(a)). Furthermore, molecular analysis was conducted using reverse transcription‐polymerase chain reaction with RNA extracted from formalin‐fixed and paraffin‐embedded tissues. This analysis revealed the presence of the *SYT‐SSX2* fusion gene (Fig. 4(b)). An *SYT‐SSX2* fusion point was also detected through sequence analysis of the polymerase chain reaction products using an automated sequencing system (Fig. 4(c)). These data led to a definitive diagnosis of monophasic spindle‐cell synovial sarcoma.

The patient has not experienced recurrence for 16 months following surgery, or occurrence of a primary tumor at any site including the lower and upper extremities.

## Discussion

The present case highlighted two important clinical issues: (i) Primary pulmonary synovial sarcoma can manifest as a nodule with calcification shown on CT; and (ii) gene analysis is useful in reaching a definitive diagnosis for this disease. In addition, we focused on radiological and histological calcification findings for primary pulmonary synovial sarcoma.

Synovial sarcoma is derived from multipotent stem cells capable of differentiating into mesenchymal and/or epithelial cells. Histologically, synovial sarcoma can be classified into the biphasic, monophasic fibrous or spindle‐cell type, and poorly differentiated type.^1^, ^4^ The vast majority (79%–88%) of synovial sarcomas arising in the lung belong to the monophasic spindle‐cell type.^3^, ^5^ Among those arising in the soft tissue, the proportion is 50%–60%.^1^ Notably, this case showed the monophasic spindle‐cell type.

The first important clinical finding is that pulmonary synovial sarcoma can manifest as a nodule with calcification shown on CT. Focal calcifications – rarely accompanied by ossification – shown on radiographs or CT scans are identified in ≤30% of cases with soft tissue synovial sarcoma.^6^, ^7^ In addition, a recent study investigating patterns of calcification demonstrated that stippled or punctate calcifications should raise suspicion regarding the presence of synovial sarcoma rather than osteosarcoma or liposarcoma.^8^ However, in primary pulmonary synovial sarcoma, intratumoral calcification is less frequently reported (15%–17%).^3^, ^9^ Although the exact reason remains unknown, this observation may be attributed to the more frequent calcification reported in the biphasic versus the monophasic type.^10^ In the present case, histological findings revealed that the thick collagen bands separating malignant spindle cells were continuous with areas of hyalinized collagen and accompanied by calcified deposits. These findings are distinctive features of a synovial sarcoma with calcification observed in cases with soft tissue synovial sarcoma. Interestingly, a recent study demonstrated the presence of punctate or amorphous calcifications shown on CT in seven patients (50%) with primary pulmonary synovial sarcoma.^5^

Regarding the imaging of lung nodules, from the CT perspective, intranodal calcifications are more commonly observed in benign lesions: inflammatory granulomas caused by mycobacterial or mycotic infections, or a benign tumor (e.g., hamartoma). In contrast, calcifications are less frequently detected in primary lung cancers (6.0%).^11^ However, among several patterns of calcium deposition (i.e., appearance and distribution), a stippled or punctate and eccentric pattern, as in the present case, tends to be observed in malignant lung nodules. In our case, the additional data of a contrast‐enhanced tumor with FDG uptake led us to perform an open lung biopsy, resulting in a completion lobectomy.

The second important clinical finding is that gene analysis was useful in the diagnosis of this disease. In the present case, we considered the presence of a synovial sarcoma based on morphological and immunohistochemical findings. However, it was necessary to differentiate this type of sarcoma from others which are categorized as malignant spindle‐cell tumors (i.e., fibrosarcoma, leiomyosarcoma, solitary fibrous tumor, and malignant peripheral nerve sheath tumors). Synovial sarcoma is characterized by the t (X;18) (p11;q11) chromosome translocation, which is exclusively observed in this tumor. The *SS18* gene on chromosome 18 and one of the *SSX* genes (*SSX1*, *SSX2*, or *SSX4*) on the X chromosome are fused, leading to the generation of the *SS18‐SSX1*, *SS18‐SSX2*, or – rarely – *SS18‐SSX4* fusion oncogenes.^5^, ^9^, ^10^, ^12^ Initially, we performed a fluorescence in situ hybridization analysis using formalin‐fixed and paraffin‐embedded tissues,^10^ which revealed an *SS18* split‐signal in almost all tumor cells. The subsequent RT‐PCT analysis demonstrated that the present case harbored the *SS18‐SSX2* fusion oncogene. Regarding the association of gene fusion and histology, almost all biphasic and monophasic types have been shown to harbor the *SS18‐SSX1* and *SS18‐SSX2* fusion oncogenes, respectively.^10^, ^12^, ^13^

Primary pulmonary synovial sarcomas are rare, comprising <0.5% of all primary lung malignancies.^5^, ^14^ However, considering the diagnostic challenges linked to this condition, an appropriate treatment plan or a definitive diagnosis may not be reached in several clinical cases. Of note, the five‐year disease‐specific survival of patients with intrathoracic synovial sarcomas is 31.6%.^9^ Moreover, larger tumor size (i.e., ≥5 cm) is a poor prognostic factor in this setting. Therefore, earlier detection of this disease may contribute to improved outcome.

In conclusion, primary pulmonary synovial sarcoma can manifest as a nodule with calcification shown on CT. In addition, gene analysis is useful in reaching a definitive diagnosis for this disease. Primary pulmonary synovial sarcoma should be listed as a differential diagnosis for solitary pulmonary nodules with punctuate and eccentric calcification. When considering synovial sarcoma based on CT findings and immunohistochemical analysis, oncology surgeons should complement the examination by performing fluorescence in situ hybridization analysis. Further investigations are warranted to determine whether a primary pulmonary synovial sarcoma with calcification may be more frequently detected.

## Disclosure

The authors report no conflict of interest.
